# Biobank linked to SWEDEHEART quality registry—routine blood sample collection opens new opportunities for cardiovascular research

**DOI:** 10.1080/03009734.2018.1498957

**Published:** 2018-09-25

**Authors:** Tomasz Baron, Anna Beskow, Stefan James, Bertil Lindahl

**Affiliations:** a Department of Medical Sciences, Cardiology, Uppsala University, Uppsala, Sweden;; b Uppsala Clinical Research Center, Uppsala, Sweden;; c Uppsala Biobank, Uppsala, Sweden

**Keywords:** Biobank, cardiovascular research, quality registry, SWEDEHEART

## Abstract

High-quality biobanking within routine health services, through the use of existing health-care practices and infrastructure, with respect to safety and integrity of patients in line with the Swedish Biobank Act, enables large-scale collection of biological material at reasonable costs. Complementing the extensive information on myocardial infarction patients from a national registry gives unique opportunities for research focusing on better understanding of cardiovascular disease occurrence and prognosis, developing of new diagnostic methods, and personalized treatments with greater efficacy and fewer side effects.

## Biobanks

The term ‘biobank’ is primarily used to describe collections of biological material of different size—from large-scale population collections to human biological specimens collected for health-care purposes. Today, the material is stored in many different ways across Sweden, ranging from large specially designed freezing rooms to single freezers spread in hospitals and research departments. Currently a lot of work is being done and has been done to transform many small biobanks into sample collections in a larger biobank organization, for example, Uppsala Biobank in Sweden.

The concept of a biobank is relatively new. The first article using this term was published by Loft and Poulsen in 1996 (1). Collection and storing of tissue samples began, however, far earlier, and already in 1858 Virchow published a work on biological samples (2). According to a survey conducted recently by the Swedish National Biobank Council, there are currently approximately 150 million blood, cell, or tissue samples collected in biobanks for care and treatment and approximately 7.5 million samples collected in biobanks for purposes of research or clinical trials (3); every year about 2–3 million new samples are stored in the Swedish biobanks (4). However, most are collected as part of a clinical trial or other temporary studies from highly selected patient groups.

## Biobank research

Biological material, such as tissue or blood samples, can be used to study genetic factors or protein expression in various diseases. A detailed phenotype description required for most research projects is commonly retrieved from patient records, registers, or questionnaires.

Samples collected primarily for health-care purposes can be used in research if the patient/donor has given consent. When the primary purpose of the sample collection is research, both an approved ethical application and an informed consent from the donors are required.

### Quality registry for coronary heart disease

The Swedish Web-system for Enhancement and Development of Evidence-based care in Heart disease Evaluated According to Recommended Therapies (SWEDEHEART) is a national quality registry for cardiovascular care, coronary angiography, percutaneous coronary intervention (PCI), cardiac surgery, secondary prevention, and cardio-genetic diseases, which was formed in 2009 by the merging of four existing registries (5). The purpose of the registry is to develop and improve cardiovascular care by continuously providing information on care needs and treatments on local, regional, and national levels. The SWEDEHEART registry and its earlier four separate registries have contributed to the enormous improvements of cardiac care and dramatic increase of survival after myocardial infarction in the last decade (6,7). However, despite the fact that diagnosis, treatment, and secondary prevention of coronary heart disease have developed favorably and that cardiac care has been improved in accordance with national guidelines, there are still shortcomings, which are reflected in, among other things, significant variations between different hospitals and regions regarding treatment regimens, with consequences for public health and health economics.

### Quality registry research

In order to achieve even better treatment results in the future, new and better knowledge about the genetic background to cardiovascular disease, new diagnostic and predictive markers, and new effective and individualized treatments are needed. Already extensive research is being carried out using data from SWEDEHEART (5), which can be escalated by linking biobank data and samples to the registry.

### Concept of a biobank linked to the SWEDEHEART registry

There are currently several regional initiatives where blood samples are collected as part of individual research projects in which phenotypic information is obtained from the SWEDEHEART registry. Cooperation between the various regional initiatives enables samples from the different biobanks to be available nationally.

Starting the collection of samples linked to the SWEDEHEART registry in the Uppsala–Örebro region aimed to create a whole workflow entirely within routine clinical care. High-quality blood samples collected from consecutive patients with myocardial infarction could be then used in future research. After approval by the Regional Ethical Review Board in Uppsala, the blood sample collection was started on 12 September 2011 at the Department of Cardiology, Uppsala University Hospital, in close collaboration with the Clinical Laboratory, Uppsala Clinical Research Center, and Uppsala Biobank.

The inclusion of patients is performed by routine health-care professionals, and the samples are collected on two occasions: first, during the hospitalization at the cardiac intensive care unit (ICU) due to the acute myocardial infarction (acute phase); and second, before or in connection with the regular follow-up visit after 6–10 weeks (stable phase). On these occasions, the information that the samples have been taken is recorded in the SWEDEHEART registry. The collected specimens are: whole blood for later DNA extraction, as well as EDTA-plasma and citrate-plasma for biomarkers specific for e.g. cardiac function, atherosclerosis, inflammation, coagulation, and renal function. Inclusion and exclusion criteria are presented in Table I.

**Table I.** The inclusion and exclusion criteria for participation in the SWEDEHEART biobank.

Inclusion criteria (all fulfilled)	Exclusion criteria (at least one of the following)
• myocardial infarction diagnosis • age ≤75 • patient included in SWEDEHEART signed informed consent	• patient transferred from another hospital • >72 hours from myocardial infarction diagnosis to planned biobank sampling • procedure-related myocardial infarction (type 4a or 5)

### Practical setup

**Figure 1.**
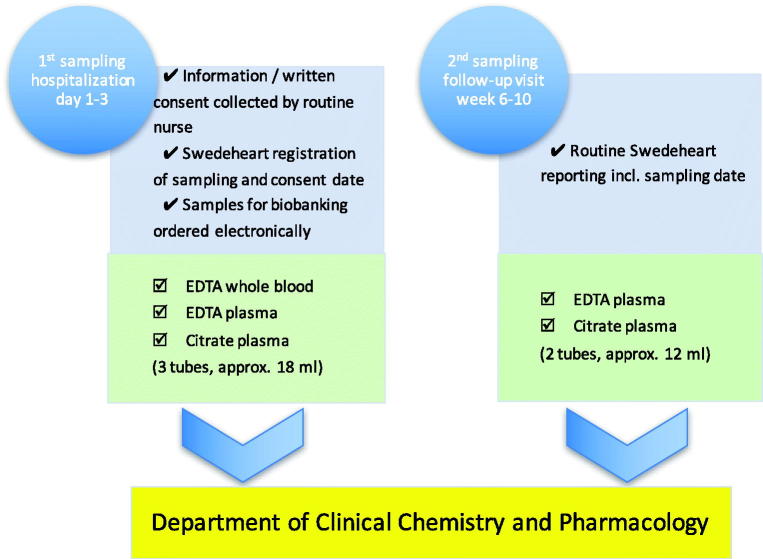
Practical setup of biobank sampling in routine care within the framework of the SWEDEHEART registry.

**Figure 2.**
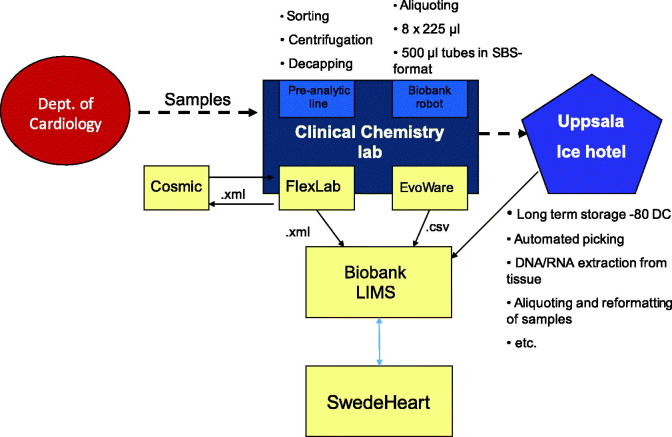
Workflow in the Clinical Chemistry and Pharmacology Laboratory, Academic Hospital. (Cosmic = patient data journal; FlexLab and EvoWare = laboratory data systems; LIMS = laboratory information management system).

Patients hospitalized at the cardiac ICU, Uppsala University Hospital, who meet criteria for inclusion in the biobank are informed about the sample collection by a routine nurse and asked for participation. Written consent is collected and scanned to be stored in the electronic patient record. Samples for biobanking are ordered electronically via the electronic patient record in the same way as, and usually with, regular samples ordered for laboratory analysis. The samples are sent to the Hospital Clinical Laboratory, Department of Clinical Chemistry and Pharmacology (KKF). At first sampling, usually the day after admission, EDTA whole blood, EDTA plasma, and citrate plasma are taken; in the second sampling after 6–10 weeks, only EDTA plasma and citrate plasma are taken. At KKF, the samples are handled and sorted by a pre-analytic robot according to the sample type specified by a four-digit extension on the tube ID. Tubes with ‘whole blood’ are registered and sorted out and frozen directly. The EDTA plasma tubes are registered, centrifuged (2400*g* for 7 min), and de-capped before being transferred to another rack for aliquoting. Citrate plasma tubes are registered and sorted into a rack for manual centrifugation, 2000*g* for 20 minutes, to ensure that it is free from platelets. Plasma from each primary tube is then transferred to eight microtubes with 225 μL each in a 96-format using a fluid-handling robot. The volume of 225 μL per microtube is more than sufficient for most modern analyses. The samples are then frozen to –80 °C and stored in Uppsala Ice Hotel (biobank premises). All information about the handling and sampling is automatically transferred from the laboratory information management system (LIMS) FlexLab together with the information from the fluid management robot to the Biobank LIMS, where complete traceability is available and information about the sample’s quality based on pre-analytical treatment is recorded. In the SWEDEHEART registry, information about consent and date of sampling is entered. The concept’s layout is presented graphically in Figures 1 and 2.

### Primary results, experiences, and strength of the concept

During the period 12 September 2011 to 31 December 2017, biobank samples were collected from a total of 534 patients of which 181 (34%) have also left samples in the second round. A detailed report one year after the start of sample collection showed that more than two-thirds of all patients with myocardial infarction who met the inclusion criteria and lacked exclusion criteria have their specimens stored in the biobank. Patients with non-ST-elevation myocardial infarction (NSTEMI) constitute 65% of the collected material, reflecting the distribution in the total infarct population. The most common reason for non-inclusion of patients appeared to be a concurrent participation in other parallel research studies, unclear initial diagnosis, lack of significant coronary artery stenosis at coronary angiography, in-hospital death before scheduled sampling, logistic reasons (transfer between departments), language deficiencies, or cognitive disorders. Out of all respondents, during the first year of sample collecting only one patient declined participation (6).

The strength of the concept is that all parts of the workflow, including sampling and specimen management, are routinely done, using existing care routines and infrastructures, and do not require additional resources in the form of special staff, which also means that the study experience is perceived as part of routine care. This means that time and resources are saved, while automated handling of samples ensures consistency and high quality. The Department of Cardiology is paid for the nurse time spent on the biobank project. The simple method that works in routine health care allows for the collection of samples of large, non-selected patient materials and where the phenotype can be described in detail at the individual level using registry data. Biobank sampling does not exclude patients from parallel participation in other research projects.

### Future development

There are ongoing efforts to enable similar biobanking in the entire health-care region. The University Hospital in Örebro is next to start the corresponding biobanking linked to the SWEDEHEART register. At the same time, work is under way with SWEDEHEART’s steering group and with researchers in other regions who are planning or have already started biobanking linked to the registry, as in the region of Skåne and Stockholm, to standardize sampling and testing, as well as to create a common set of rules for sampling. The biobank material, together with the registry’s possibilities for careful phenotyping and follow-up, will provide a unique national resource for future research.
